# Draft Whole-Genome Sequences for Two *Pigmentiphaga* Isolates Recovered from Human Clinical Materials

**DOI:** 10.1128/genomeA.00726-17

**Published:** 2017-08-03

**Authors:** Anne-Marie Bernier, Kathryn Bernard

**Affiliations:** a Department of Biology, Université de Saint-Boniface, Winnipeg, Canada; b National Microbiology Laboratory, CSCHAH Site, Public Health Agency of Canada, Winnipeg, Canada; c Department of Medical Microbiology, University of Manitoba, Winnipeg, Canada

## Abstract

Features from two *Pigmentiphaga* isolates referred to Canada’s National Microbiology Laboratory from human clinical materials were described previously (N. Bridger, S. Drews, T. Burdz, D. Wiebe, A. L. Pacheco, B. Ng, and K. Bernard, J Med Microbiol 62:708–711, 2013, https://doi.org/10.1099/jmm.0.051615-0). Whole-genome sequencing was performed on strains NML030171 and NML080357; the sequences were found to have 5.86 and 5.73 Mb of clean data and G+C contents of 67.5 and 66.74 mol%, respectively.

## GENOME ANNOUNCEMENT

The genus *Pigmentiphaga* of the family *Alcaligenaceae*, order *Burkholderiales*, class *Betaproteobacteria*, has three species (*P. daeguensis*, *P. kullae*, and *P. litoralis*) (http://www.bacterio.net/pigmentiphaga.html), all derived from the environment. “*Pigmentiphaga soli*” has been effectively described but not validly named to date (1). In 2013, we outlined features of two *Pigmentiphaga*-like isolates (National Microbiology Laboratory [NML] identifiers NML030171 and NML080357), recovered from human clinical materials; these were closest by 16S rRNA gene sequencing to the type strains of *P. daeguensis* (99.5% to 99.8% identity) and *P. kullae* (99.0 to 99.3% identity), as well as to each other (99.7% identity) (2). We provide here the draft whole-genome sequences (WGS) for these isolates, as there do not appear to be any WGS data for *Pigmentiphaga* among *Burkholderiales* genomes available for study in the public domain.

DNA was extracted using the TruSeq DNA high-throughput (HT) sample preparation kit. Paired-end whole-genome shotgun libraries were constructed using the TruSeq Nano DNA HT library preparation kit. Sequencing was performed using a MiSeq sequencer (Illumina) and yielded 2,011,338 and 2,041,846 reads for NML030171 and NML080357, respectively. Assembly was performed with SPAdes (version 3.5) using k-mer values of 21 to 127, following the merging of paired-end reads from shorter fragments using Fast Length Adjustment of SHort reads (FLASH). Assembled contigs were filtered to remove short (≤1-kb) and repeat sequences, generating draft genomes of 57 and 59 contigs with 5,862,145 and 5,734,860 bp, respectively. The G+C contents were 67.5 and 66.74%, respectively, with 5,552 and 5,427 genes. The genomes were annotated using the National Center for Biotechnology Information (NCBI) Prokaryotic Genome Annotation Pipeline (PGAP) (https://www.ncbi.nlm.nih.gov/genome/annotation_prok).

The genomes were compared to each other using JSpeciesWS to calculate the average nucleotide identity values using BLASTN (ANIb) (3). Using this approach, the two strains had ANIb scores of 84% to each other, values that are well below the suggested cutoff threshold of 94 to 96% (3), indicating that these may represent two putative new separate species. *In silico* DNA-DNA hybridization analysis (genome-to genome distance calculator [4]) supports this conclusion, with values of 50.6% using formula 1 and 30% using formula 2.

Strain NML080357 harbors one intact 21.5-kb phage composed of 24 proteins with homology to *Pseudomonas* phage JBD93. Strain NML030171 does not harbor any complete phage sequences; however, both strains have 4 clustered regularly interspaced short palindromic repeat (CRISPR) sequences. The whole-genome sequences from *Pigmentiphaga* species are not available in public databases; therefore, we could not compare our strains to the type strains of *Pigmentiphaga daeguensis* or *Pigmentiphaga kullae*.

### Accession number(s).

Draft whole-genome projects for NML030171 and NML080357 have been deposited at DDBJ/EMBL/GenBank under accession numbers NINX00000000 and NINW00000000, and the versions described in this paper are NINX01000000 and NINW01000000, respectively.
